# Impact of Simulation Based Learning on Knowledge and Skills Among Medical Students Undergoing Competency Based Medical Education

**DOI:** 10.7759/cureus.88749

**Published:** 2025-07-25

**Authors:** Devendra Prasad KJ, Rajesh K, Krisna Moorthy DGSR, Nikhil Reddy Y, Aravind S R

**Affiliations:** 1 Emergency Medicine, Sri Devaraj Urs Medical College, Kolar, IND; 2 Emergency Medicine, Sri Devaraj Urs Medical College,, Kolar, IND

**Keywords:** competency based medical education, simulation in medical education, skills and simulation training, teaching by simulation, undergraduate medical students career motivation simulation-based education immersive learning educational technology

## Abstract

Introduction

Medical education is shifting toward experiential, competency-based approaches that emphasize clinical reasoning and practical skills. Simulation-based learning (SBL) supports this shift by offering a safe, structured setting where students enhance cognitive and psychomotor abilities through active practice, timely feedback, and reflection. This fosters deeper engagement with clinical content and builds confidence before patient encounters.

Global research indicates that SBL enhances various competencies, but Indian studies are limited. Factors like insufficient early clinical exposure and high student-to-patient ratios necessitate local research. This study assesses the effects of simulation-based medical education (SBME) on clinical competencies in Indian undergraduates and investigates learner perceptions and performance influencers.

Methodology

An interventional study was performed in 2023-2024 with 56 undergraduate medical students in Phases II and III. Simulation training occurred in two phases, spaced six months apart, utilizing low- and medium-fidelity mannequins alongside validated checklists. Phase I measured performance in basic life support (BLS), advanced trauma life support (ATLS), and anterior nasal packing before and after simulation. Phase II assessed skill retention via simulations of nasogastric tube insertion and urinary catheterization, emphasizing pre-procedural steps and communication.

Results

Simulation-based training yielded significant enhancements across all five clinical procedures. In BLS, all 17 tasks demonstrated improvement (χ²=4.083-21.043, p<0.05), reflecting increased emergency response proficiency with moderate to large effect sizes (Cramer’s V=0.30-0.55). Anterior nasal packing showed progress in 11 of 13 tasks, particularly in communication and pain management, although insertion steps exhibited minimal change due to high baseline proficiency. ATLS training resulted in enhancements in 22 of 25 tasks (χ²=4.000-17.053, p<0.05; Cramer’s V=0.28-0.50), especially in airway management and documentation. Non-significant alterations in three tasks indicate potential areas for curricular enhancement. Nasogastric tube insertion demonstrated improvements in 15 of 17 tasks, particularly in procedural sequence, with moderate effect sizes (Cramer’s V=0.32-0.46). In Foley catheterization, 13 of 15 tasks improved, particularly in recognizing clinical indications (χ²=4.167, p=0.041; Cramer’s V=0.31).

Confidence intervals for effect sizes ranged from 95% CI [0.20-0.60], reflecting a consistent educational impact. Overall, simulation training significantly enhanced clinical skills, particularly in areas with moderate baseline proficiency.

Conclusion

Simulation-based learning significantly improved both immediate clinical performance and long-term skill retention, particularly in procedures with moderate baseline proficiency. Regular, structured simulations enhanced technical accuracy and patient-centered competencies within the competency based medical education (CBME) framework. While the single-center design and small sample size (n=56) limit generalizability, this reflects the institutional batch size. Despite this, the use of validated checklists ensures reliable outcomes, supporting the integration of simulation into routine medical training.

## Introduction

The progression of medical education, propelled by advancements in technology, has profoundly altered the methodologies through which both graduate and postgraduate learners assimilate knowledge and interact with their respective domains [1].

This shift is motivated by the need to prepare healthcare professionals with the skills required to manage the complexities of modern medical practice. Simulation tools now allow learners to interact with realistic clinical scenarios, enhancing decision-making and problem-solving without risking patient safety [2].

Digital platforms, virtual simulations, and mobile applications have further enriched education by offering interactive, immersive, and flexible learning experiences. These innovations have increased student engagement and supported a move toward learner-centered and interdisciplinary approaches [3,4].

Medical simulation, in particular, replicates real clinical environments to improve educational outcomes. It reduces medical errors, strengthens teamwork and communication among healthcare providers, and builds trust within healthcare systems. By allowing clinicians to practice both routine and rare scenarios in a controlled setting, simulation helps refine communication skills that are critical for effective patient care [5].

A key benefit of simulation lies in its support for enactive learning-knowledge gained through active engagement and interaction with the environment. This form of learning is often more intuitive and applicable than abstract instruction. Simulation exercises, followed by debriefing, create purposeful, hands-on experiences that improve knowledge retention and lead to positive behavioral changes in clinical practice.

## Materials and methods

Study design & setting 

A prospective observational study was conducted in the department of emergency medicine. The data collection for this study was conducted between 2023 and 2024.

Inclusion criteria

Undergraduate medical students currently enrolled in the clinical phase (typically third year or higher) of the Bachelor of Medicine, Bachelor of Surgery (MBBS) program who provided informed consent to participate in the study, and students with no prior formal training in the specific simulation procedures, were included in the study (to ensure baseline comparability).

Exclusion criteria

The exclusion criteria included participants who did not provide informed consent or withdrew from the study at any stage, students who missed either the pre-test or post-test evaluations, and students with known physical or cognitive impairments that could significantly affect their ability to perform psychomotor tasks in the simulation setting.

Ethics statement

This study was approved by the institutional human ethics committee and institutional review board of Sri Devaraj Urs Medical College (reference number: SDUMC/KLR/IEC/357/2023-24). Written informed consent was obtained from all participants after explaining the study’s purpose, procedures, and voluntary nature. Participation had no impact on academic evaluation, and confidentiality was strictly maintained.

Sample size

A total of 56 undergraduate students participated in the study as per the inclusion and exclusion criteria.

Study protocol

This study examined the impact of simulation-based learning on the clinical performance of medical students within a competency based medical education (CBME) framework by using validated checklists. The simulation assessments were conducted in two distinct phases to evaluate both immediate and delayed learning outcomes. In the first phase, pre- and post-simulation performance was assessed for basic life support (BLS), advanced trauma life support (ATLS), and nasal packing. In the second phase, conducted a few months later, nasogastric tube insertion and urinary catheterization were reassessed to specifically evaluate the retention of pre-procedural formalities (e.g., consent, analgesia) and post-procedural communication.

Statistical analysis

Descriptive analysis was carried out by frequency and percentage for categorical variables. McNemar’s test was used to test the statistical significance of cross-tabulation between paired categorical variables. P-value <0.05 was considered statistically significant. R-Studio Desktop (Posit Software, PBC, Boston, MA) was used for data analysis. 

Measurement of key outcome variables and data analysis

BLS simulations showed significant improvement in all task completions post-training (p<0.05), indicating enhanced emergency response and coordination. In nasal packing, while basic steps were consistently performed pre- and post-simulation, other components improved significantly (p<0.05), reflecting better procedural understanding after the intervention.

In the ATLS domain, most tasks demonstrated statistically significant improvement after simulation training (p<0.05), except for three tasks-external hemorrhage control by compression, administration of IV crystalloids, and ordering of chest X-rays-which showed improvement but did not reach statistical significance (p>0.05). These outcomes suggest that while simulation enhances critical trauma management skills, certain tasks may require more practice or exposure for meaningful improvement.

The second phase, conducted after a few months, was designed to assess whether the students retained not only procedural knowledge but also acquired competence in communication, consent, and post-procedural patient engagement, which are integral to patient-centered care. For nasogastric (NG) tube insertion, three core pre-procedural communication tasks-confirming patient identity, addressing pain, and obtaining consent-were already performed by all students at both time points, indicating a strong baseline understanding and retention. Other tasks, such as tube fixation and post-insertion reassurance, showed slight improvement post-simulation but were not statistically significant. This may suggest either a high baseline performance or limited reinforcement over time.

Similarly, for urinary catheterization, most technical and aseptic practices were consistently performed by all participants at both time points. A statistically significant improvement was observed in recognizing the indication for Foley catheterization (p<0.05), highlighting that simulation reinforced clinical decision-making. However, tasks such as securing the catheter and checking tubing for kinks, although improved, did not show statistical significance, possibly due to ceiling effects. Interestingly, minor lapses were observed in maintenance-related practices (e.g., collection bag placement), indicating that certain routine practices may decline without regular reinforcement.

All simulation sessions were standardized using pre-validated procedural checklists and conducted in a dedicated simulation lab to ensure consistency. Facilitators underwent prior training in simulation delivery and debriefing techniques to maintain uniformity in instruction and assessment. A control group was not included due to logistical constraints; instead, each participant served as their own control through pre- and post-intervention assessments.

Low- to medium-fidelity mannequins and task trainers were used, depending on the procedure. Assessments were conducted by trained faculty members using validated checklists. While blinding was not feasible due to the instructional format, efforts were made to ensure consistency by providing assessor training, and checklist-based scoring helped reduce subjectivity.

## Results

Phase one results

**Figure 1:**
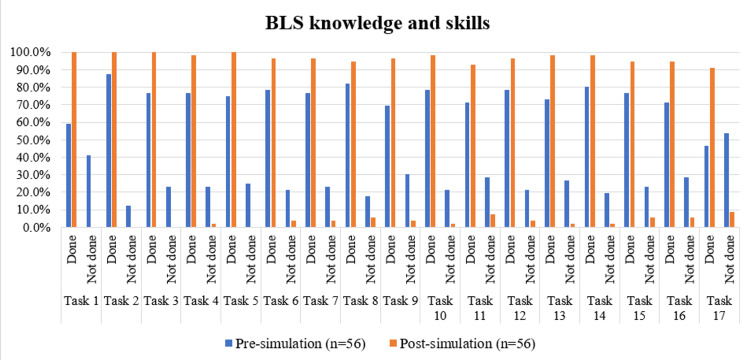
Comparison of BLS knowledge and skills pre and post simulation-based learning programme Tasks for basic life support (BLS), from 'check scene safety' to ‘team discusses the events '', have been sequentially coded as Task 1 to Task 17 for data visualization. Marked improvement was observed in critical steps such as checking scene safety, calling for help, pulse and breathing check, activating the emergency response system, automated external defibrillator (AED) use, ensuring chest recoil, resuming cardiopulmonary resuscitation (CPR) after shock, airway management using the E-C clamp technique, appropriate ventilation timing, and team communication. These results underscore the effectiveness of simulation in reinforcing essential resuscitation skills.

As depicted in Table 1 and Figure 1, for all the BLS tasks, there was a statistically significant increase in task completion at the post-simulation time point compared to the pre-simulation time point (p<0.05). This was determined using McNemar’s test, with corresponding chi-squared (χ²) values ranging from 4.083 to 21.043. Phase one results of BLS, anterior nasal packing, and trauma life support were done by using checklists shown in Appendices 1, 2, 3.

**Table 1:** Comparison of BLS knowledge and skills pre and post simulation-based learning program BLS: basic life support, AED: automated external defibrillator, CPR: cardiopulmonary resuscitation, C/V: compression ventilation ratio.

Task	Done/Not done	Time		P-value
		Pre (n=56)	Post (n=56)	
Check scene safety	Done	33 (58.9%)	56 (100%)	<0.001*
	Not done	23 (41.1%)	0 (0%)	
Check response	Done	49 (87.5%)	56 (100%)	0.016
	Not done	7 (12.5%)	0 (0%)	
Call for help	Done	43 (76.8%)	56 (100%)	<0.001*
	Not done	13 (23.2%)	0 (0%)	
Check for carotid pulse and breathing for 5-10 seconds	Done	43 (76.8%)	55 (98.2%)	<0.001*
	Not done	13 (23.2%)	1 (1.8%)	
Activate the emergency response system and get an AED	Done	42 (75%)	56 (100%)	<0.001*
	Not done	14 (25%)	0 (0%)	
Hand placement over the lower half of the sternum with a compression rate of 100-120/min and depth of 5-6cm	Done	44 (78.6%)	54 (96.4%)	0.002
	Not done	12 (21.4%)	2 (3.6%)	
Allow complete recoil	Done	43 (76.8%)	54 (96.4%)	0.001*
	Not done	13 (23.2%)	2 (3.6%)	
Minimize interruptions for less than 10 seconds and maintain a 30:2 C/V ratio with breath over 1 second	Done	46 (82.1%)	53 (94.6%)	0.016
	Not done	10 (17.9%)	3 (5.4%)	
Switch on the AED and follow the commands	Done	39 (69.6%)	54 (96.4%)	<0.001*
	Not done	17 (30.4%)	2 (3.6%)	
Avoid contact with the victim while analysing the rhythm and delivering the shock	Done	44 (78.6%)	55 (98.2%)	0.001
	Not done	12 (21.4%)	1 (1.8%)	
Resume CPR immediately after delivering shock	Done	40 (71.4%)	52 (92.9%)	<0.001*
	Not done	16 (28.6%)	4 (7.1%)	
Position at the head end of the patient	Done	44 (78.6%)	54 (96.4%)	0.002
	Not done	12 (21.4%)	2 (3.6%)	
Use the E-C clamp technique for holding the device, head tilt, and chin lift for opening the airway	Done	41 (73.2%)	55 (98.2%)	<0.001*
	Not done	15 (26.8%)	1 (1.8%)	
Compress only half of the bag	Done	45 (80.4%)	55 (98.2%)	0.002
	Not done	11 (19.6%)	1 (1.8%)	
Avoid excessive ventilation	Done	43 (76.8%)	53 (94.6%)	0.002
	Not done	13 (23.2%)	3 (5.4%)	
1 breath every 6 seconds once an advanced airway is in place.	Done	40 (71.4%)	53 (94.6%)	<0.001*
	Not done	16 (28.6%)	3 (5.4%)	
Team discusses the events	Done	26 (46.4%)	51 (91.1%)	<0.001*
	Not done	30 (53.6%)	5 (8.9%)	

**Figure 2:**
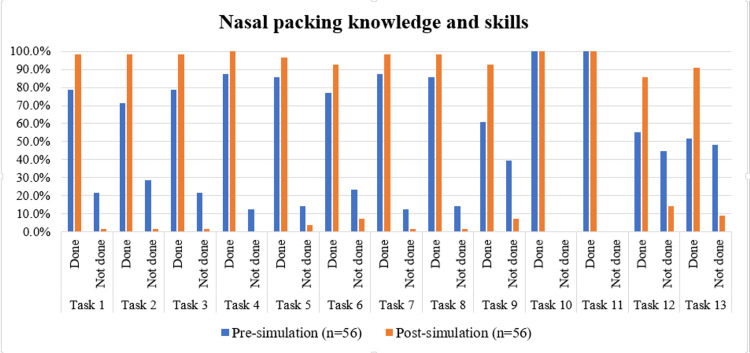
Comparison of nasal packing knowledge and skills pre and post simulation-based learning program Tasks for nasal packing, from 'confirm identity of the patient' to 'address patients concerns and reassure’, have been sequentially coded as Task 1 to Task 13 for data visualization.

As depicted in Table 2 and Figure 2, the comparison of task performance before and after simulation for anterior nasal packing revealed statistically significant improvements in several key areas, as determined by McNemar’s test. Significant improvements (p<0.05) were observed in tasks such as addressing pain (χ²=10.562, p=0.001), addressing patient concerns and providing reassurance (χ²=9.481, p=0.002), warning the patient prior to inserting the nasal pack (χ²=7.682, p=0.006), confirming patient identity (χ²=6.750, p=0.009), obtaining consent (χ²=6.750, p=0.009), and identifying the indication for nasal packing (χ²=5.143, p=0.023).

Other tasks, such as the use of antibiotic-coated nasal packs, knotting the pack threads, positioning the patient upright, use of sterile gloves, and performing complete hand hygiene, did not show statistically significant changes (p>0.05), although improvements were noted numerically.

**Table 2:** Comparison of nasal packing knowledge and skills pre and post simulation-based learning program

Task	Done/Not done	Time		P-value
		Pre (n=56)	Post (n=56)	
Confirm identity of the patient	Done	44 (78.6%)	55 (98.2%)	0.001
	Not done	12 (21.4%)	1 (1.8%)	
Address pain	Done	40 (71.4%)	55 (98.2%)	<0.001*
	Not done	16 (28.6%)	1 (1.8%)	
Consent from patient/family members	Done	44 (78.6%)	55 (98.2%)	0.001
	Not done	12 (21.4%)	1 (1.8%)	
Student identifies the indication of anterior nasal packing	Done	49 (87.5%)	56 (100%)	0.016
	Not done	7 (12.5%)	0 (0%)	
Performs all steps of hand hygiene before and after anterior nasal packing	Done	48 (85.7%)	54 (96.4%)	0.031
	Not done	8 (14.3%)	2 (3.6%)	
Use sterile gloves and appropriate antiseptic solution	Done	43 (76.8%)	52 (92.9%)	0.004
	Not done	13 (23.2%)	4 (7.1%)	
Position patient in upright sitting position	Done	49 (87.5%)	55 (98.2%)	0.031
	Not done	7 (12.5%)	1 (1.8%)	
Use single use packet of antibiotic coated nasal pack for insertion	Done	48 (85.7%)	55 (98.2%)	0.016
	Not done	8 (14.3%)	1 (1.8%)	
Warn the patient that you are about to insert the pack and ask him to breath through mouth	Done	34 (60.7%)	52 (92.9%)	<0.001*
	Not done	22 (39.3%)	4 (7.1%)	
Insert both nasal packs through patient nostrils	Done	56 (100%)	56 (100%)	-
	Not done	0 (0%)	0 (0%)	
Anterioposteriorly inflate both the packs by wetting them with normal saline	Done	56 (100%)	56 (100%)	-
	Not done	0 (0%)	0 (0%)	
Knot both the threads of the pack	Done	31 (55.4%)	48 (85.7%)	<0.001*
	Not done	25 (44.6%)	8 (14.3%)	
Address patients concerns and reassure	Done	29 (51.8%)	51 (91.1%)	<0.001*
	Not done	27 (48.2%)	5 (8.9%)	

**Figure 3:**
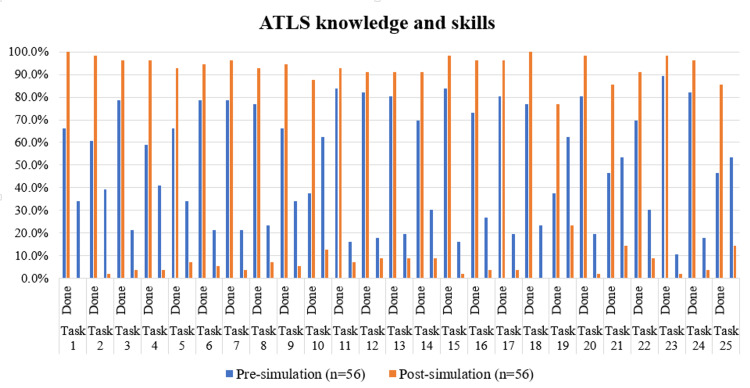
Comparison of ATLS knowledge and skills pre and post simulation-based learning program Tasks for advanced trauma life support (ATLS), from 'confirm identity of the patient' to ' perform secondary survey for further evaluation’, have been sequentially coded as Task 1 to Task 25 for data visualization. Notable improvements were observed in confirming patient identity, addressing pain, applying manual in-line stabilization, using the jaw thrust maneuver, performing needle decompression for tension pneumothorax, sealing open pneumothorax, applying a pelvic binder, documenting Glasgow Coma Scale (GCS), identifying focal neurological deficits, preventing hypothermia, performing a logroll, obtaining an electrocardiogram (ECG), and completing the secondary survey. These findings emphasize the effectiveness of simulation-based training in enhancing rapid assessment, airway and hemorrhage control, neurological evaluation, and critical interventions in trauma care.

As shown in Table 3 and Figure 3, McNemar’s test demonstrated a statistically significant improvement in several critical trauma care tasks post-simulation. These included confirming patient identity (χ²=17.053, p<0.001), addressing pain (χ²=16.409, p<0.001), manual in-line stabilization or application of a cervical collar (χ²=14.087, p<0.001), performing a three-way seal for open pneumothorax (χ²=11.429, p=0.001), identifying focal neurological deficits (χ²=11.077, p=0.001), needle decompression (χ²=7.579, p=0.006), documentation of GCS (χ²=6.667, p=0.010), use of blood warmer and blankets (χ²=5.818, p=0.016), logroll and secondary survey (both χ²=5.633, p=0.018), jaw thrust maneuver (χ²=5.263, p=0.022), symmetric chest rise and airway patency check (both χ²=4.083, p=0.043), and limb immobilization with E-FAST (χ²=4.000, p=0.046).

**Table 3:** Comparison of ATLS knowledge and skills pre and post simulation-based learning program ATLS: advanced trauma life support, SpO2: peripheral capillary oxygen saturation, ICS: intercostal space, MTP: massive transfusion protocol, PRBC: packed red blood cells, GCS: Glasgow Coma Scale, ECG: electrocardiogram, EFAST: extended focused assessment with sonography for trauma.

Task	Done/Not done	Time		P-value
		Pre (n=56)	Post (n=56)	
Confirm identity of the patient	Done	37 (66.1%)	56 (100%)	<0.001*
	Not done	19 (33.9%)	0 (0%)	
Address pain	Done	34 (60.7%)	55 (98.2%)	<0.001*
	Not done	22 (39.3%)	1 (1.8%)	
Check for airway patency (able to finish full sentences)	Done	44 (78.6%)	54 (96.4%)	0.002
	Not done	12 (21.4%)	2 (3.6%)	
Manual in line stabilization/head block/c-collar application	Done	33 (58.9%)	54 (96.4%)	<0.001*
	Not done	23 (41.1%)	2 (3.6%)	
Use jaw thrust maneuver to open airway	Done	37 (66.1%)	52 (92.9%)	<0.001*
	Not done	19 (33.9%)	4 (7.1%)	
Advanced airway when indicated	Done	44 (78.6%)	53 (94.6%)	0.004
	Not done	12 (21.4%)	3 (5.4%)	
Look for symmetric chest rise	Done	44 (78.6%)	54 (96.4%)	0.002
	Not done	12 (21.4%)	2 (3.6%)	
O2 support if SPO2 <94%	Done	43 (76.8%)	52 (92.9%)	0.004
	Not done	13 (23.2%)	4 (7.1%)	
Needle decompression in 5th ICS for tension pnuemothorax	Done	37 (66.1%)	53 (94.6%)	<0.001*
	Not done	19 (33.9%)	3 (5.4%)	
3-way seal in case of open pnuemothorax	Done	21 (37.5%)	49 (87.5%)	<0.001*
	Not done	35 (62.5%)	7 (12.5%)	
External hemorrhage control by compression for 5-10 mts	Done	47 (83.9%)	52 (92.9%)	0.063
	Not done	9 (16.1%)	4 (7.1%)	
IV crystalloids for loss of blood	Done	46 (82.1%)	51 (91.1%)	0.063
	Not done	10 (17.9%)	5 (8.9%)	
Arrange for PRBC/Activate MTP in massive blood loss	Done	45 (80.4%)	51 (91.1%)	0.031
	Not done	11 (19.6%)	5 (8.9%)	
Pelvic binder application in case of pubic symphysis diastasis	Done	39 (69.6%)	51 (91.1%)	<0.001*
	Not done	17 (30.4%)	5 (8.9%)	
Immobilizing the limb in case of long bone fracture and e fast examination to r/o internal bleeding	Done	47 (83.9%)	55 (98.2%)	0.008
	Not done	9 (16.1%)	1 (1.8%)	
Document GCS	Done	41 (73.2%)	54 (96.4%)	<0.001*
	Not done	15 (26.8%)	2 (3.6%)	
Look for pupillary reaction and anisocoria	Done	45 (80.4%)	54 (96.4%)	0.004
	Not done	11 (19.6%)	2 (3.6%)	
Look for any focal neurological deficit	Done	43 (76.8%)	56 (100%)	<0.001*
	Not done	13 (23.2%)	0 (0%)	
Prevent hypothermia	Done	21 (37.5%)	43 (76.8%)	<0.001*
	Not done	35 (62.5%)	13 (23.2%)	
Use a blood warmer and warm blankets in case of hypothermia	Done	45 (80.4%)	55 (98.2%)	0.002
	Not done	11 (19.6%)	1 (1.8%)	
Logroll	Done	26 (46.4%)	48 (85.7%)	<0.001*
	Not done	30 (53.6%)	8 (14.3%)	
ECG	Done	39 (69.6%)	51 (91.1%)	<0.001*
	Not done	17 (30.4%)	5 (8.9%)	
Chest X-ray	Done	50 (89.3%)	55 (98.2%)	0.063
	Not done	6 (10.7%)	1 (1.8%)	
EFAST	Done	46 (82.1%)	54 (96.4%)	0.008
	Not done	10 (17.9%)	2 (3.6%)	
Performs secondary survey for further evaluation	Done	26 (46.4%)	48 (85.7%)	<0.001*
	Not done	30 (53.6%)	8 (14.3%)	

Phase two results

**Figure 4:**
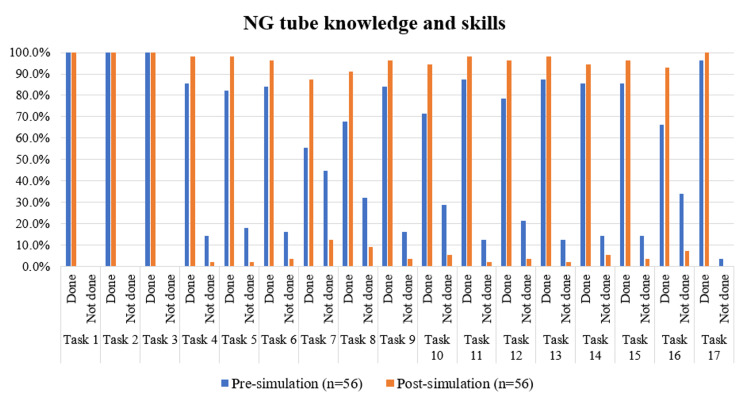
Comparison of NG tube knowledge and skills pre and post simulation-based learning program Tasks for nasogastric (NG) tube, from 'confirm identity of the patient' to 'address patients concerns and reassure’, have been sequentially coded as Task 1 to Task 17 for data visualization.

The pre-test and post-test for nasogastric (NG) tube procedures, as mentioned in Table 4 and Figure 4, were conducted a few months after those for nasal packing, BLS, and ATLS. This was done to ensure that students didn't overlook important pre-procedural procedures like confirming the patient's identity, getting consent, and administering analgesia. The goal was to demonstrate how simulation-based training helps aspiring physicians provide comfort care by improving their communication skills and procedural knowledge. Hence, all the pre-procedural tasks for the NG tube, i.e., confirm identity of the patient, address pain, and consent from patient/family member, were done by all 56 participants at both time points (pre and post). Phase two results of NG tube insertion and urinary catheterization were done using checklists, as shown in Appendices 4, 5.

**Table 4:** Comparison of NG tube knowledge and skills pre and post simulation-based learning program NG: nasogastric.

Task	Done/Not done	Time		P-value
		Pre (n=56)	Post (n=56)	
Confirm identity of the patient	Done	56 (100%)	56 (100%)	-
	Not done	0 (0%)	0 (0%)	
Address pain	Done	56 (100%)	56 (100%)	-
	Not done	0 (0%)	0 (0%)	
Consent from patient/family members	Done	56 (100%)	56 (100%)	-
	Not done	0 (0%)	0 (0%)	
Student identifies the indication of rules tube insertion	Done	48 (85.7%)	55 (98.2%)	0.016
	Not done	8 (14.3%)	1 (1.8%)	
Performs all steps of hand hygiene before and after rules tube insertion	Done	46 (82.1%)	55 (98.2%)	0.004
	Not done	10 (17.9%)	1 (1.8%)	
Position the patient upright with head in neutral position	Done	47 (83.9%)	54 (96.4%)	0.016
	Not done	9 (16.1%)	2 (3.6%)	
Measure from bridge of the nose to ear lobe and then 5cm below xiphisternum	Done	31 (55.4%)	49 (87.5%)	<0.001*
	Not done	25 (44.6%)	7 (12.5%)	
Use sterile gloves and lubricate the tip of NG tube	Done	38 (67.9%)	51 (91.1%)	<0.001*
	Not done	18 (32.1%)	5 (8.9%)	
Use single use packet of lubricant jelly for each patient	Done	47 (83.9%)	54 (96.4%)	0.016
	Not done	9 (16.1%)	2 (3.6%)	
Warn the patient that you are about to insert NG tube	Done	40 (71.4%)	53 (94.6%)	<0.001*
	Not done	16 (28.6%)	3 (5.4%)	
Insert NG tube through one of the patient Nostril	Done	49 (87.5%)	55 (98.2%)	0.031
	Not done	7 (12.5%)	1 (1.8%)	
Advice the patient to swallow and not to cough once NG tube reaches pharynx	Done	44 (78.6%)	54 (96.4%)	0.002
	Not done	12 (21.4%)	2 (3.6%)	
Continue to advance the NG tube down the oesophagus	Done	49 (87.5%)	55 (98.2%)	0.031
	Not done	7 (12.5%)	1 (1.8%)	
Once the desired insertion length is reached, NG tube is fixed	Done	48 (85.7%)	53 (94.6%)	0.063
	Not done	8 (14.3%)	3 (5.4%)	
Check tube position	Done	48 (85.7%)	54 (96.4%)	0.031
	Not done	8 (14.3%)	2 (3.6%)	
Attempt to aspirate gastric contents	Done	37 (66.1%)	52 (92.9%)	<0.001*
	Not done	19 (33.9%)	4 (7.1%)	
Address patients concerns and reassure	Done	54 (96.4%)	56 (100%)	0.500
	Not done	2 (3.6%)	0 (0%)	

There was a statistically significant improvement in the performance of several nasogastric (NG) tube insertion tasks following the simulation-based intervention, as assessed by McNemar’s test. Significant gains were observed in tasks such as measuring the nose-ear-xiphisternum length (χ²=9.031, p=0.003), attempting to aspirate gastric contents (χ²=8.522, p=0.004), warning the patient before NG insertion (χ²=7.579, p=0.006), using sterile gloves and lubricating the tip (χ²=6.261, p=0.012), performing hand hygiene before and after the procedure (χ²=5.818, p=0.016), advising the patient to swallow once the tube reached the pharynx (χ²=5.786, p=0.016), and identifying the indication for Ryle’s tube insertion (χ²=4.000, p=0.046).

On the other hand, tasks such as proper positioning of the patient, use of single-use lubricant, insertion and advancement of the NG tube, checking tube position, and fixation of the NG tube at the desired length demonstrated statistically insignificant increments in task completion at the post-simulation time point (p>0.05). For the final task-"Address patient’s concerns and reassure", although completion improved from 54/56 pre-simulation to 56/56 post-simulation, the marginal increase of just two completions was insufficient to reach statistical significance (p=0.480).

**Figure 5:**
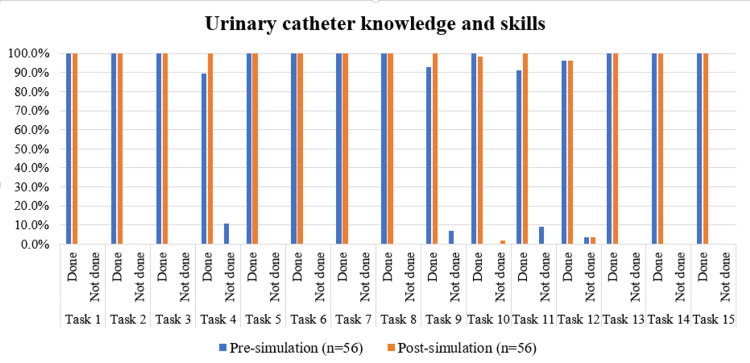
Comparison of urinary catheter knowledge and skills pre and post simulation-based learning program Tasks for urinary catheter, from 'confirm identity of the patient' to 'address patients concerns and reassure’, have been sequentially coded as Task 1 to Task 15 for data visualization.

As per Table 5 and Figure 5, the comparison of task performance related to Foley’s catheterisation before and after the simulation revealed a statistically significant improvement in the task “Student identifies the indication of Foley’s catheterisation” (χ²=4.167, p=0.041), as assessed using McNemar’s chi-squared test. Since this simulation module was conducted a few months after the previous sessions on nasal packing, BLS, and ATLS, all pre-procedural tasks-including confirming patient identity, addressing pain, and obtaining consent-were performed by all 56 participants at both time points. Similarly, all participants consistently completed other procedural steps, such as hand hygiene, use of sterile gloves and antiseptics, aseptic cleaning technique, use of single-use lubricant, maintenance of a closed drainage system, regular emptying of the collecting bag, and addressing patient concerns.

**Table 5:** Comparison of urinary catheter knowledge and skills pre and post simulation-based learning program

Task	Done/Not done	Time		P-value
		Pre (n=56)	Post (n=56)	
Confirm identity of the patient	Done	56 (100%)	56 (100%)	-
	Not done	0 (0%)	0 (0%)	
Address pain	Done	56 (100%)	56 (100%)	-
	Not done	0 (0%)	0 (0%)	
Consent from patient/family members	Done	56 (100%)	56 (100%)	-
	Not done	0 (0%)	0 (0%)	
Student identifies the indication of foley's catheterisation	Done	50 (89.3%)	56 (100%)	0.031
	Not done	6 (10.7%)	0 (0%)	
Performs all steps of hand hygiene before and after foley's insertion	Done	56 (100%)	56 (100%)	-
	Not done	0 (0%)	0 (0%)	
Use sterile gloves and appropriate antiseptic solution/sterile water	Done	56 (100%)	56 (100%)	-
	Not done	0 (0%)	0 (0%)	
Use aseptic technique, clean genitals from centre to periphery	Done	56 (100%)	56 (100%)	-
	Not done	0 (0%)	0 (0%)	
Use single use packet of lubricant jelly for each patient	Done	56 (100%)	56 (100%)	-
	Not done	0 (0%)	0 (0%)	
Secure catheter to prevent movement and urethral traction	Done	52 (92.9%)	56 (100%)	0.125
	Not done	4 (7.1%)	0 (0%)	
Keep collection bag below the level of bladder all times	Done	56 (100%)	55 (98.2%)	1.000
	Not done	0 (0%)	1 (1.8%)	
Check tubing frequently for kinking	Done	51 (91.1%)	56 (100%)	0.063
	Not done	5 (8.9%)	0 (0%)	
Keep drainage bag off the floor	Done	54 (96.4%)	54 (96.4%)	1.000
	Not done	2 (3.6%)	2 (3.6%)	
Empty the collecting bag regularly	Done	56 (100%)	56 (100%)	-
	Not done	0 (0%)	0 (0%)	
Maintain a closed drainage system	Done	56 (100%)	56 (100%)	-
	Not done	0 (0%)	0 (0%)	
Address patients concerns and reassure	Done	56 (100%)	56 (100%)	-
	Not done	0 (0%)	0 (0%)	

For the tasks “Secure catheter to prevent movement and urethral traction” (χ²=2.250, p=0.134) and “Check tubing frequently for kinking” (χ²=3.200, p=0.074), although task completion improved from 52/56 and 51/56, respectively, at pre-simulation to 56/56 at post-simulation, these increases were not statistically significant (p>0.05). In the task “Keep collection bag below the level of bladder at all times”, performance slightly declined from 100% (56/56) at pre-simulation to 98.2% (55/56) post-simulation (χ²=0.000, p=1.000). Similarly, changes in performance for “Keep drainage bag off the floor” (χ²=0.250, p=0.617) were minimal and not statistically significant, with two participants failing to meet this criterion post-simulation despite improvements from two others.

Key factors enhancing cognitive and psychomotor skills in simulation training: student feedback analysis

Factors responsible for improving cognitive and psychomotor skills among medical students in simulation-based scenarios were analyzed after taking feedback from students at the end of the study under three categories as follows: 

a. Prior knowledge variables

b. Teaching methodology variables and 

c. Outcome variables

A detailed representation of these categorized student responses is provided in Appendix 6.

As mentioned in Table 6, a substantial proportion of participants (91%) did not consider prior knowledge of the subjects essential before their involvement in the simulation program, with only a negligible segment (8.9%) responding 'Maybe,' and none claiming it to be a requisite. Upon inquiring about their acquaintance with the subjects before the simulation, 76.7% disclosed that they were not at all acquainted, whereas only 12.5% and 10.7% categorized themselves as very familiar and familiar, respectively. It is noteworthy that none of the participants possessed prior experience with simulation-based training, as 100% indicated no such exposure. These results imply that the participant cohort predominantly consisted of individuals engaging in simulation for the first time with scant prior knowledge, thereby emphasizing the significance of simulation as a formidable introductory pedagogical instrument in clinical training.

**Table 6:** Prior knowledge in the subject (N=56)

Prior Knowledge Variables	Frequency	Percentage
Do you think prior knowledge about the topics is must before attending simulation programme?		
Yes	0	0.0%
Maybe	5	8.9%
No	51	91.0%
How familiar are you about the topics before simulation?		
Very familiar	7	12.5%
Familiar	6	10.7%
Nil familiar	43	76.7%
Do you have experience in attending simulation?		
Yes	0	0.0%
Maybe	0	0.0%
No	56	100.0%

As mentioned in Table 7 of Teaching methodology, the responses reflect a robustly favourable perception of the simulation teaching methodology among the participants. A significant majority (89.2%) indicated that the prior provision of clinical materials was instrumental in adequately preparing them for the simulation, and 98.2% concurred that the establishment of clear objectives facilitated their concentration on the content effectively. All participants (100%) expressed that the pre-briefing session enhanced their comfort levels before the commencement of the simulation, thereby underscoring the critical role of orientation in alleviating anxiety and fostering readiness. Moreover, 87.5% regarded the simulated procedures as realistic and analogous to authentic patient scenarios, suggesting a high degree of fidelity within the simulation experience. Notably, 94.6% of participants recognized that debriefing proved beneficial in reinforcing the skills acquired during the session. These findings underscore the efficacy of structured simulation components-such as preparation, clarity of objectives, realism, and reflective debriefing- augmenting the overall learning experience.

**Table 7:** Teaching methodology (N=56)

Teaching methodology Variables	Frequency	Percentage
Prior supply of clinical material helped you before attending simulation?		
Yes	50	89.2%
Maybe	6	10.8%
No	0	0.0%
Clear objectives helped you to focus on the content?		
Yes	55	98.2%
Maybe	1	1.8%
No	0	0.0%
Pre briefing made you comfortable?		
Yes	56	100.0%
Maybe	0	0.0%
No	0	0.0%
Do you feel the procedures in simulation are as realistic as patients?		
Yes	49	87.5%
Maybe	7	12.5%
No	0	0.0%
Do you agree that debriefing is useful in aggregating the learned skill?		
Yes	53	94.6%
Maybe	3	5.4%
No	0	0.0%

As mentioned in Table 8 for outcome variables, data reveal overwhelmingly positive perceptions regarding the impact of simulation-based education on clinical competence and professional development. A near-total majority (98.2%) agreed that simulation programs can enhance team performance, while 94.6% believed that such programs also improve communication skills, critical components of effective clinical practice. Notably, all participants (100%) affirmed that regular participation in simulation increases their confidence in real-world clinical settings, underscoring the value of repeated exposure for skill consolidation. Furthermore, 96.4% agreed that simulation-based education enables the timely achievement of clinical competencies. These findings strongly support the role of simulation as a powerful, confidence-building, and competency-enhancing educational strategy within medical training.

**Table 8:** Outcome (N=56)

Outcome Variables	Frequency	Percentage
Do you think simulation programmes can improve team performance?		
Yes	55	98.2%
Maybe	1	1.8%
No	0	0.0%
Do you think simulation programmes can improve your communication?		
Yes	53	94.6%
Maybe	3	5.4%
No	0	0.0%
Do you agree that regular participation in simulation programmes can bring more confidence in your clinical practice?		
Yes	56	100.0%
Maybe	0	0.0%
No	0	0.0%
Do you agree hat competencies for clinical practice are achievable within time by simulation-based education?		
Yes	54	96.4%
Maybe	2	3.6%
No	0	0.0%

## Discussion

The findings of this study demonstrate that structured, phase-wise simulation-based learning significantly enhances clinical performance across a range of essential emergency and procedural skills in undergraduate medical education. By adopting a longitudinal, multi-skill design spaced over six months, this study uniquely captures both immediate learning gains and long-term skill retention - an approach that addresses the limitations of previous research, which often focuses on single procedures or short-term outcomes.

Statistically significant improvements were observed across five core domains: BLS, ATLS, anterior nasal packing, nasogastric tube insertion, and urinary catheterization. Notably, tasks with moderate baseline proficiency, such as team coordination in BLS, neurological assessment in ATLS, and patient communication in nasal packing, showed the most pronounced improvement (p<0.001), underscoring the dual value of simulation in reinforcing both technical skills and non-technical competencies like communication, teamwork, and patient interaction.

The novelty of this study lies in its dual-phase simulation structure, use of validated procedural checklists, and exclusive focus on undergraduate learners within a CBME framework. While earlier studies primarily support the role of SBL in postgraduate training, our findings extend its relevance to early clinical education.

Foundations of experiential learning

Edgar Dale’s cone of experience constitutes a seminal framework that categorizes learning experiences along a spectrum from direct, hands-on engagement to progressively abstract representations, including verbal symbols. It highlights the assertion that knowledge retention is markedly enhanced when learners participate actively in experiential activities, such as simulations, demonstrations, or real-world involvement, as opposed to passive modalities like reading or auditory instruction [6].

This framework is intricately aligned with Kolb’s experiential learning theory, which articulates that effective learning is a cyclical process encompassing four distinct stages: concrete experience, reflective observation, abstract conceptualization, and active experimentation [7]. Both paradigms emphasize the pivotal role of active engagement in cultivating profound comprehension and enduring retention. In contemporary educational contexts that prioritize learner-centered approaches and multimedia integration, these theories retain considerable significance. Simulation-based education, for example, epitomizes the synthesis of Dale’s and Kolb’s principles-providing learners with tangible experiences that incite reflection, conceptual development, and iterative practice, thereby ultimately augmenting clinical proficiency and decision-making capabilities in authentic environments.

Simulation in healthcare education: theoretical perspectives

As David Gaba emphasizes, simulation should be viewed not merely as a technological tool but as a robust pedagogical methodology that replicates real-world clinical experiences within a safe, interactive environment to improve learning and patient safety [8]. Drawing on Dale’s learning pyramid, Gaba frames simulation as a versatile educational strategy applicable across diverse healthcare settings. His model further classifies simulation across 11 key dimensions, including learner level, domain of care, simulation purpose, and feedback approach, advocating for its systematic incorporation into healthcare training and operations to enhance safety and clinical outcomes.

Complementing Gaba’s perspective, the Simnovate Engaged Learning Domain Group offers a contemporary framework that emphasizes three pivotal dimensions of simulation-based pedagogy: scope, modality, and environment [9]. This structure provides a more nuanced understanding of how simulation should be integrated into health professions education, especially in terms of fidelity and learner engagement. By breaking down simulation activities into these interconnected dimensions, the Simnovate model aims to transform acquired knowledge and procedural training into real-world clinical proficiency-thereby closing the gap between simulation performance and actual patient care.

Together, these frameworks reinforce the value of our study's design and outcomes, suggesting that thoughtfully implemented, spaced, and multi-dimensional simulation training is a powerful driver of competency development in undergraduate medical education. This evidence strengthens the argument for embedding simulation as a core, recurring component of CBME curricula to promote sustained clinical excellence.

Scope encompasses the complexity of the simulated clinical scenario, closely tied to defined learning objectives. Simulations can vary from simple tasks like IV cannulation to complex emergencies requiring collaboration and decision-making. Scope is vital for targeting competencies, advanced procedures, and non-technical skills.

Modality pertains to the simulation tools' type and fidelity, which affect realism in learning experiences. Fidelity measures the simulation's resemblance to real clinical environments; low-fidelity tools serve early skill development, while high-fidelity options enhance immersion for complex scenarios. Selecting the right modality depends on session goals, resources, and learner training levels.

The environment of simulation includes various settings, from controlled labs to real clinical scenarios and virtual platforms. Each setting provides distinct advantages. In situ simulations enhance realism and teamwork but necessitate careful planning and safety. Classroom simulations create a safe environment conducive to skill development and feedback. Virtual simulations present flexibility and accessibility for remote participants [10].

These three dimensions collectively establish a framework for educators to design engaging and goal-oriented simulation experiences. This model promotes strategic planning of simulation activities to ensure learners acquire practical and transferable skills within an immersive context.

Simulation-based medical education (SBME): linking theory to practice

SBME bridges classroom learning with clinical practice by creating dynamic environments for skill acquisition without patient risk. It aligns with Experiential Learning Theory and Adult Learning Theory, which emphasize hands-on experience, reflection, and real-world problem-solving [11].

Effective SBME improves clinical reasoning, technical skills, and learner confidence through active practice and timely feedback. Integrating specific, measurable, achievable, relevant, time-bound (SMART) learning objectives into existing curricula can significantly enhance student learning outcomes by providing clear, structured, and actionable goals. This integration ensures that educational programs are aligned with desired learning outcomes, facilitating better planning, assessment, and student engagement [12].

Building competency through simulation

Medical curricula must ensure students acquire core competencies, including communication, professional behavior, physical examination skills, diagnostic and therapeutic decision-making, and teamwork [13].

Simulation-based learning has a profound impact on the development of non-technical skills among healthcare professionals, particularly in high-pressure clinical situations. These skills, which include communication, teamwork, decision-making, and stress management, are crucial for effective clinical practice and patient safety. SBL provides a controlled, risk-free environment where healthcare professionals can practice and refine these skills, leading to improved clinical outcomes and enhanced professional confidence [14].

The manuscript authored by Shrivastava et al. underscores the significance of debriefing and feedback within the realm of SBME. It asserts that although engagement in simulations facilitates the learning process, the provision of effective feedback is paramount for the attainment of educational objectives [15].

Debriefing sessions provide educators with the opportunity to evaluate students' knowledge, competencies, and attitudes, which subsequently impact their overall performance. The authors conclude that the strategic planning of feedback sessions and the training of educators in these competencies are imperative for the enhancement of learning outcomes in medical education programs.

SBME provides an immersive and interactive learning environment that encourages self-directed learning. Learners can engage with realistic clinical scenarios, allowing them to practice and refine their skills independently, and it is particularly effective for younger, tech-savvy learners familiar with interactive, game-based platforms [16].

The significance of sociological fidelity within interprofessional simulation lies in its ability to nurture patient-centered care among healthcare trainees, as it promotes an understanding of professional duties, strengthens communication, and fosters collaborative engagement [17]. This methodology underscores the social dynamics and interactions that transpire in authentic healthcare environments, which are vital for the efficacy of patient-centered care. Through the simulation of these interactions, learners are allowed to gain a deeper comprehension of the intricacies associated with teamwork and communication, ultimately resulting in enhanced patient outcomes.

Study rationale and relevance to Indian medical education

While global studies affirm the value of simulation-based learning, research within the Indian medical education context remains sparse. This study investigates the effectiveness of SBME in enhancing clinical knowledge and skills among undergraduate medical students in India.

SBME has shown particular benefits for learners with limited prior clinical exposure [18]. Collaborative learning environments within SBME promote enhanced communication skills and increased engagement [19]. Empirical studies support the use of adaptable group structures revised continuously through ongoing evaluation as a strategy to boost learner interaction and communication proficiency. This dynamic setup also facilitates the inclusion of diverse viewpoints, enriching problem-solving capabilities, and deepening conceptual understanding through peer-to-peer interaction [20].

Structured yet flexible modules that extend beyond the core curriculum further reinforce these outcomes. Such learning experiences foster teamwork, presentation abilities, and academic curiosity. Students benefit from the opportunity to explore real-world scenarios collaboratively, thereby strengthening both subject-specific knowledge and essential soft skills. Feedback from participants underscores notable improvements in communication, collaboration, and the application of learned concepts in clinical contexts, affirming the value of integrative, student-centered approaches within SBME [21].

The observed advancements in both the swift acquisition of competencies and the enduring retention over time through simulation-based pedagogical methods in this study align with the principles of experiential learning. Yardley et al. (2012) expound upon Kolb’s seminal framework, highlighting the conversion of tangible clinical experiences into profound learning through processes of reflection, conceptualization, and active experimentation, an approach that is particularly conducive to the systematic cycles inherent in simulation-based training [22].

In this investigation, the simulation exercises presented authentic clinical scenarios that enabled learners to partake in practical experiences, followed by structured debriefing, thereby facilitating all components of Kolb’s learning cycle. These interactive, contextually rich experiences are particularly vital for the mastery of intricate emergency procedures and communication tasks, as evidenced by the performance outcomes of the participants.

Moreover, these results align with established frameworks in simulation-based education that emphasize curriculum coherence, systems integration, and innovation. The structured and iterative characteristics of the simulations employed in this study are consistent with key features identified by Issenberg et al. (2005), such as deliberate practice, feedback, and contextual relevance. By embedding simulation within an evidence-based paradigm and aligning it with clinical requirements and learner progression, this methodology fosters both immediate competence and long-term skill retention critical for high-stakes environments like emergency medicine [23].

These findings in this study not only reaffirm the pedagogical value of simulation in developing a broad spectrum of clinical competencies but also highlight its adaptability for undergraduate medical education when integrated within a structured, competency-based framework. Given the sustained improvements observed across multiple domains and the strong learner feedback, the study supports the case for wider implementation of longitudinal simulation programs in early clinical training.

## Conclusions

This study highlights the importance of simulation-based learning as a crucial component of CBME, demonstrating measurable improvements in both immediate skill acquisition and long-term retention across a range of essential emergency procedures. The longitudinal and structured methodology-prioritizing spaced assessments, intentional practice, and systematic feedback, demonstrated efficacy in fostering enduring competence and the amalgamation of both technical and non-technical skills.

Simulation significantly enhanced procedural adeptness in high-stakes scenarios such as BLS, ATLS, and anterior nasal packing, whereas deferred evaluations in nasogastric tube insertion and urinary catheterization accentuated its role in cultivating patient-centered care and enduring clinical reasoning. These findings advocate for the systematic incorporation of simulation at regular intervals within the medical curriculum to facilitate both procedural mastery and professional growth.

In light of these findings, we advocate for the incorporation of SBL into the undergraduate MBBS curriculum to establish robust clinical foundations. Regular refresher sessions and structured debriefings ought to be integrated throughout the training process to uphold competencies and promote reflective learning.

Nevertheless, this study is not without limitations. It was conducted at a single institution with a relatively small sample size and a short follow-up duration, which may limit the generalizability and long-term interpretation of the findings. The absence of a control group and the potential for assessor bias further constrain the internal validity of the results. Additionally, variations in individual learning styles and preferences-particularly relevant in online and hybrid learning contexts, were not explicitly accounted for, which may have influenced learner engagement and outcomes. Despite these limitations, the focused design allowed for consistent instructional delivery and close monitoring of learner progress, providing a controlled environment to evaluate the intervention’s feasibility and impact. The findings offer a replicable framework that can be adapted to institutions with similar educational settings. Future research involving multi-center studies with larger, more diverse cohorts is essential to validate these results and assess scalability, contextual flexibility, and long-term educational impact, particularly in low- and middle-income countries.

## Figures and Tables

**Figure 6:**
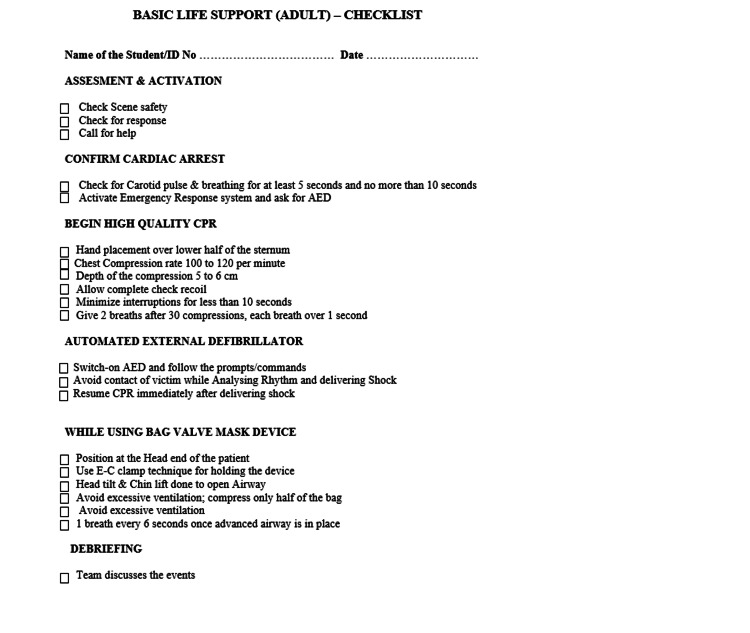
Basic life support (adult) – checklist

**Figure 7:**
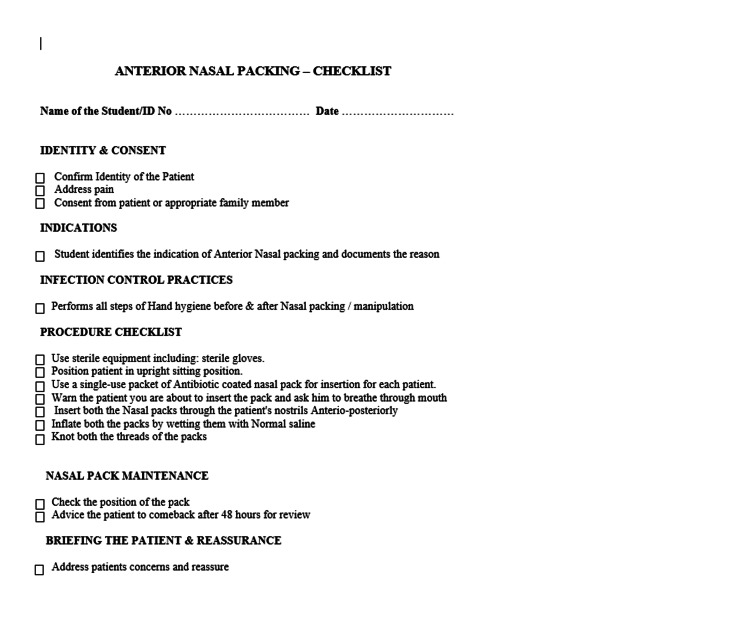
Anterior nasal packing - checklist

**Figure 8:**
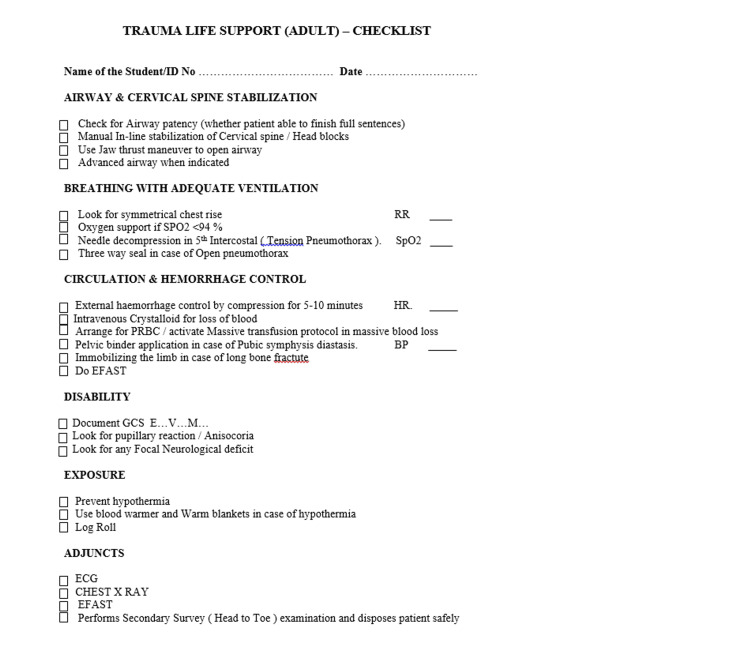
Trauma life support - checklist

**Figure 9:**
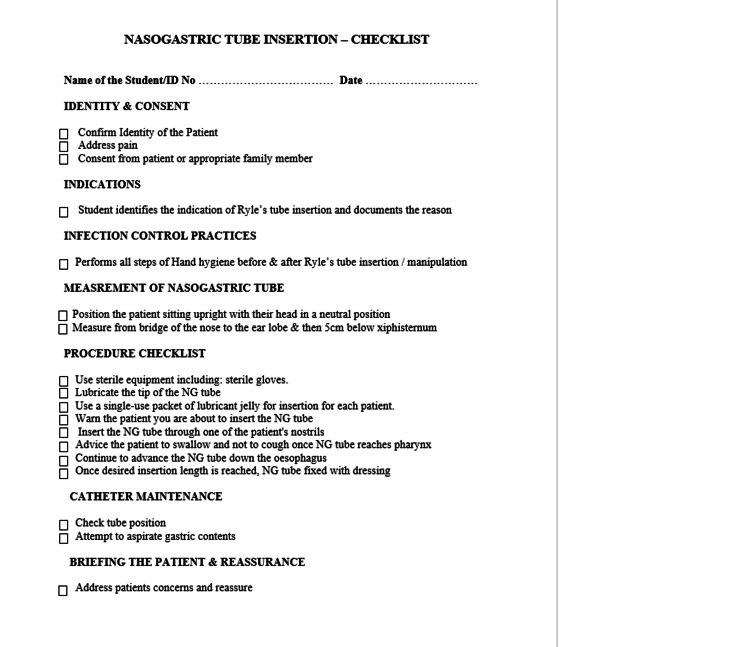
Nasogastric tube insertion - checklist

**Figure 10:**
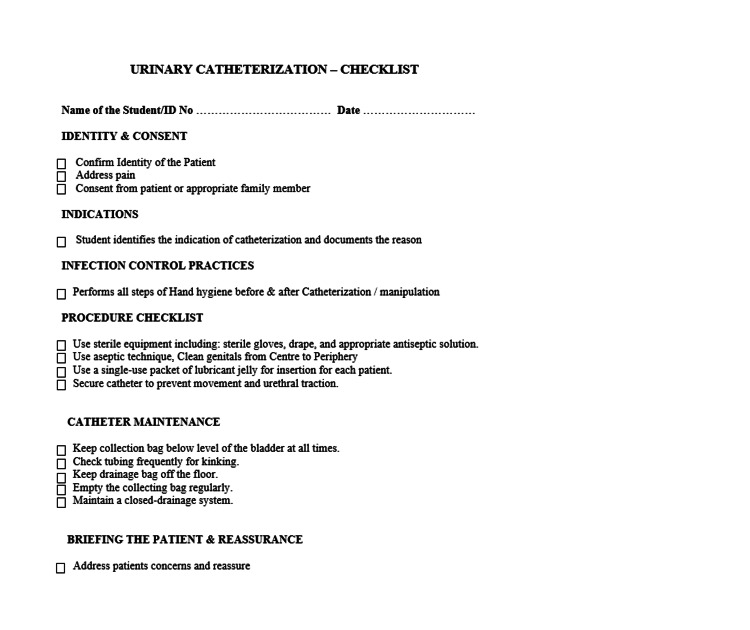
Urinary catheterization - checklist

**Figure 11:**
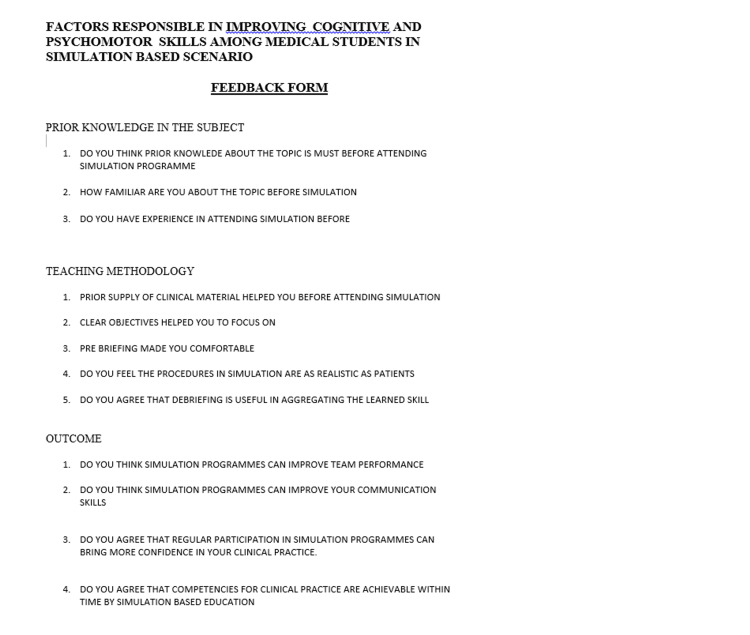
Feedback form

## Appendices

Appendix 1 

Appendix 2 

Appendix 3 

Appendix 4 

Appendix 5 

Appendix 6 
